# Knowledge of HIV status in a rural Ugandan population with a sustained, generalised HIV epidemic

**DOI:** 10.1371/journal.pgph.0007354

**Published:** 2026-10-01

**Authors:** Keith Tomlin, Ronald Makanga, Janet Seeley, Emma Slaymaker, Robert Newton, Joseph Mugisha

**Affiliations:** 1 Department of Population Health, London School of Hygiene & Tropical Medicine, United Kingdom; 2 MRC/UVRI and LSHTM Uganda Research Unit, Entebbe, Uganda; 3 Department of Global Health and Development, London School of Hygiene & Tropical Medicine, United Kingdom; University of California Irvine, UNITED STATES OF AMERICA

## Abstract

For people living with HIV, knowledge of their status is essential to access antiretroviral therapies (ART). This knowledge can be measured by population surveys that record self-reported HIV status before offering HIV testing. We conducted a survey among residents of 25 villages in the rural sub-county of Kyamulibwa in Southwest Uganda where there has been a sustained, generalised HIV epidemic. Through the survey we determined the proportions of those living with HIV who knew their status (with reference to UNAIDS targets), exposures associated with knowledge of HIV status, and the proportions of those who knew their HIV status who reported taking ART. Data were collected from June 2019 to July 2021. Among survey participants, 39.7% of men and 28.7% of women had never tested for HIV. This was associated with older age, male sex, lower education and never having been married. All participants consented to HIV testing and 99.9% had a result. 83.6% of men and 88.0% of women living with HIV already knew their status. Of these, 99% of men and women reported taking ART. Of those never previously tested, 2.5% of men and 2.7% of women received a positive diagnosis. HIV prevalence was 8.1% among men and 10.5% among women. While significant proportions of survey participants did not know their HIV status they did not represent a large source of undiagnosed HIV infection. The proportions of men and women living with HIV who already knew their status were below current UNAIDS targets to end AIDS by 2030, and the study highlighted groups for whom participation in testing needs to increase. Among those with HIV who knew their status almost all reported taking ART, suggesting high acceptance and good availability of treatment. The overall prevalence of HIV among survey participants means that reliable access to sufficient ART is essential.

## Introduction

Voluntary testing for HIV remains the cornerstone of global HIV management and control. Individuals who don’t know that they are living with HIV cannot benefit from antiretroviral therapy (ART) that, if consistently supplied and taken as directed, can substantially reduce the morbidity and mortality that would otherwise arise from HIV infection [1]. ART can also significantly reduce onward transmission of the virus [2] and has contributed to falling HIV incidence in many settings [3]. WHO guidelines now state that anyone diagnosed with HIV should commence ART as soon as possible after receiving their positive test result [4].

The critical importance of HIV testing was underscored by its inclusion in the “90-90-90” targets set by the Joint United Nations Programme on HIV/AIDS in 2014 [5]. These stated that, by 2020, 90% of all those living with HIV would know of their diagnosis, 90% of those diagnosed would be receiving sustained ART and that, of these, 90% would achieve sufficient virological suppression to maintain immunological health. In September 2020 UNAIDS reported “good progress” in global attainment of these targets [6] although distinct regional and demographic inequalities in this progress had been identified [7]. On 8^th^ June 2021 the United Nations General Assembly released a political declaration on HIV and AIDS that retained the three targets but raised the thresholds to 95-95-95, to be achieved by 2030 as part of a strategy to eliminate AIDS as a public health threat by this date [8]. These updated targets also introduced more nuanced measures of achievement with specific objectives relating to key populations of sex workers, transexual people, men who have sex with men, people who inject drugs, and those in prisons and other confined locations [8]. These objectives were based on evidence that key populations are disproportionately living with HIV whilst simultaneously receiving poorer HIV care, or no care at all [9].

Accurate assessment of population HIV testing can be challenging [9] and the failure to do so significant – in the United States between 2010 and 2014 it was estimated that undiagnosed HIV was responsible for one-third of all new HIV acquisitions [10]. Numerous methods of assessing HIV testing have been applied and among these are population surveys that include the offer of an HIV test to all participants [11]. This approach has the advantages that self-reported knowledge of HIV status can measured, the proportion of undiagnosed HIV infection can be estimated, progress towards targets can be clearly calculated and social, demographic and sexual behaviour data can identify the characteristics of those who have been reluctant to test.

The greatest impact of HIV continues to be in Eastern and Southern Africa where, in 2023, 450,000 (360,000–580,000) people with newly acquired HIV were identified [12]. In 2021, 91% (83%-98%) of those aged ≥15 who were living with HIV in Eastern and Southern Africa were estimated to know of their diagnosis [13], but this broad estimate can conceal a wide range of national and regional variation. Demographic and Health Surveys (DHS) and Health & Demographic Surveillance Systems (HDSS) in Africa have revealed that adult men, younger and older ages of both sexes and those with less than primary-level education are among those less likely to know they are living with HIV [14]. Such surveys need to be frequently repeated to capture the evolving dynamics of generalised HIV epidemics where access to treatment and care can change rapidly.

In 2021 it was estimated that 1.4 [1.3–1.5] million people aged ≥15  in Uganda were living with HIV, of whom 81% were aware of their diagnosis [15]. National HIV prevalence in 2021 was estimated to be 5.2% [16,17], but this ranged from 0.3% in Nabilatuk in the North East to 16.2% in parts of the Buganda region in the south (15). In 2021 74% of the population live in rural areas [18] and the decentralisation of HIV services in 2012, while increasing access to HIV testing and care in remote areas, has been inconsistently applied and measured [19]. Social pressure, stigma and aggressive discrimination against some key populations [20,21] have also contributed to late presentation for testing and care. HIV epidemiology in Uganda is therefore characterised by powerful local determinants of infection, institutionalised deterrents to care and fragmented service provision and measurement. This means that local and frequent estimation of HIV testing is required if undiagnosed HIV infection is not to fuel the epidemic in some areas once again.

The primary aims of this study were a) to determine the self-reported knowledge of HIV status in a rural population in Southwest Uganda that has experienced a sustained and generalised HIV epidemic; b) to determine the proportion of those living with HIV in this population who knew their status (the “first 95” of the UNAIDS targets); and c) to assess demographic and behavioural exposures that may be associated with knowledge of HIV status in this population. Secondary aims of the study were to update the HIV prevalence in the population and to determine, among those participants who knew that they were living with HIV, the proportion taking ART. The study addressed these aims by conducting a cross-sectional medical survey in which participants were asked to self-report demographic and behavioural characteristics and their knowledge of HIV status before being offered an HIV test.

## Methods

### Ethics statement

Ethical approval for this study was granted by the UVRI Research and Ethics Committee (GC/127/19/10/710) and the Uganda National Council for Science and Technology (SS 4981). Ethical approval from the UVRI was first granted for 12 months on 12^th^ April 2019 and was subsequently extended via continuous review to 15^th^ May 2026. Ethical approval from the UNCST was first granted on 16^th^ May 2019 for 24 months and was subsequently extended via continuous review to 16^th^ May 2028. All study participants were required to give written informed consent, and parental consent was also required for those participants aged <18 years, in addition to assent from the minor.

### Study setting and population

This study was undertaken by the MRC/UVRI and LSHTM Uganda Research Unit, details of which have been given extensively elsewhere [22]. A key component of the Unit is the Kyamulibwa Health and Demographic Surveillance System (HDSS), also known as the General Population Cohort (GPC), which comprises 25 contiguous villages, with a total population of approximately 25,000 residents, in the rural sub-county of Kyamulibwa in the Kalungo district of Southwest Uganda. The GPC was established in 1989 in response to some of the first cases of AIDS being identified in the area [23] and since then it has helped to characterise the epidemiology of HIV throughout what has become a sustained, generalised epidemic in a rural East African setting. Data collection within the GPC includes an annual census of all residents with a detailed recording of residency, births, deaths and migration. These censuses are accompanied by biannual medical surveys that focus upon different themes such as HIV diagnoses and sexual behaviour. As well as providing a setting for data collection the GPC also hosts a clinic that offers free healthcare to residents, including the provision of ART. Through a programme of care decentralisation by the Ugandan Ministry of Health from 2012 a further three public health centres now provide HIV testing and care services within the GPC area.

### Data collection methods

Data collection for this study comprised two stages. The first was a new census of all households in the GPC to define the study population. A mapping exercise was first carried out to establish the number and location of all occupied households within the GPC. Census enumerators then visited each household with the aim of interviewing the household head and other household members who were present. Interviewees were asked for written consent to be interviewed and – where this was given – were asked about demographic characteristics and changes in residency and their family structure. Household heads gave information on changes to household occupancy since the previous census. Census responses were entered directly by the interviewers into Microsoft Access databases hosted on laptops running Microsoft Windows 7. A random 10% sample of households was selected for re-interview by a different enumerator to monitor data quality and interviewer variability.

These new census data were then used to define the study population for the second stage of data collection, a medical survey. Approximately two weeks after the census had been completed in a village, a house was rented in that village by the survey team from which the survey was conducted. All men aged ≥18 and women aged ≥16 who were currently resident in the village were eligible for the survey and invited to take part. The survey team held contact details collected during the census of all eligible participants and would prompt them up to four times to voluntarily participate in the survey. Participation comprised four steps – requesting written informed consent, collecting data on a structured questionnaire, taking biophysical measurements and collecting blood and urine samples. Participants could give partial consent if they wished to exclude themselves from the final step.

In the structured questionnaire survey participants were asked about their current HIV status and their use of ART if this was applicable. All participants, regardless of self-reported status, were offered an HIV rapid test (Abbott Determine, STAT-PACK or Uni-gold) in accordance with Ugandan Ministry of Health guidelines. A non-fasting venous blood sample was also collected from each participant on which ELISA or Western Blot tests could be conducted if two rapid tests returned either a positive or an indeterminate result. Survey interview data were again entered directly into Microsoft Access databases.

### Analytical methods

Individual-level data from the census, survey and laboratory records were linked via a unique person ID number. Linking the census and survey records enabled an analysis of survey participation to reveal any selection biases that might arise from voluntary survey engagement. Individual-level characteristics analysed in association with survey participation were sex, age, religion, highest level of education, marital status and time spent away from home. For survey participants the associations were then assessed between these exposures, plus alcohol and tobacco use, and knowledge of HIV status. As the single round of census data and the medical survey were both cross-sectional datasets, these associations were assessed using logistic regression that calculated odds and odds ratios in multivariate models where each exposure was adjusted for all others. For each outcome the association with sex was determined, after which the results for men and women were presented separately to reveal differences within sex. The role of each exposure was assessed using likelihood ratio tests and a random effects term was included in all regression models to account for clustering within households.

The results from the HIV tests conducted during the survey were then compared to those from self-reported HIV status, to calculate those who already knew that they were living with HIV and those who received a positive HIV result for the first time. All data were analysed using Stata 17 (StataCorp USA).

### Inclusivity in global research

Additional information regarding the ethical, cultural, and scientific considerations specific to inclusivity in global research is included in the Supporting Information (S1 Checklist)

## Results

### Survey participation

The census and medical survey were both conducted between 28th June 2019 and 7th July 2021. Data collection was interrupted between 11th March and 20th October 2020 due to movement restrictions arising from the Covid-19 pandemic. In the 25 villages that form the GPC a total of 4,704 households were visited by the census enumerators and 24,894 individual census records were updated.

For this analysis the eligible study population for the survey was all adults aged ≥ 18 years who were identified by the census as being resident in one of the 25 GPC villages during the enumeration period. Two individuals were excluded due to missing data for one of the study exposures. 8,473 people were included in the eligible study population of whom 3,770 (44.5%) were men, with a mean age of 39.1 years (SD 17.0). The mean age of women was 40.3 years (SD 17.7).

2,471 men (65.5% of those eligible) and 3,731 women (79.3% of those eligible) volunteered to take part in the medical survey. In a multivariable analysis, with each exposure adjusted for all others, the overall odds of women participating in the survey were over twice those of men (adjusted odds ratio (aOR) 2.19, 95% confidence interval (95%CI) 1.94-2.48, p < 0.0001). Table 1 describes, for men and women separately, the associations between survey participation and age, religion, education, marital status and time spent away from the household.

**Table 1:** Survey participation from eligible census population.

	Participated/Total Eligible N (%)		*Adjusted Odds Ratio p-value (LRT)* Adjusted Odds Ratio	
Exposures	Men	Women	Men	Women
**Age group**			***<0.0001***	***<0.0001***
18 – 24	525/951 (55.2)	752/1112 (67.6)	1.00	1.00
25 – 34	506/806 (62.8)	778/984 (79.1)	1.10 (0.84-1.45)	1.32 (1.02-1.70)
35 – 44	441/689 (64.0)	777/884 (87.9)	1.06 (0.77-1.45)	2.47 (1.81-3.37)
45 – 54	455/621 (73.2)	586/689 (85.1)	1.68 (1.20-2.36)	1.99 (1.43-2.77)
55+	544/703 (77.4)	838/1034 (81.0)	2.02 (1.43-2.84)	1.53 (1.12-2.10)
**Religion**			***0.6626***	***0.9972***
Christian	1862/2824 (65.9)	2779/3509 (79.2)	1.00	1.00
Muslim	609/946 (64.4)	952/1194 (79.7)	0.96 (0.79-1.16)	1.00 (0.82-1.21)
**Highest level of education**			***0.1006***	***<0.0001***
None/primary	1620/2407 (67.3)	2453/2985 (82.2)	1.00	1.00
Secondary	662/1024 (64.7)	1050/1358 (77.3)	1.09 (0.90-1.32)	1.00 (0.82-1.22)
Post-secondary	189/339 (55.8)	228/360 (63.3)	0.78 (0.59-1.04)	0.52 (0.38-0.70)
**Marital status**			***<0.0001***	***<0.0001***
Never married	664/1200 (55.3)	549/854 (64.3)	1.00	1.00
Currently married	1418/2088 (67.9)	2034/2416 (84.2)	1.48 (1.15-1.90)	2.20 (1.71-2.84)
Divorced or widowed	389/482 (80.7)	1148/1433 (80.1)	2.77 (1.93-3.97)	1.47 (1.09-1.98)
**Time spent in household last year**			***0.1804***	***0.0012***
Normally resident	2028/3000 (67.6)	3046/3723 (81.8)	1.00	1.00
6 to 12 months in household	333/564 (59.0)	480/669 (71.8)	0.82 (0.65-1.03)	0.67 (0.53-0.84)
0 to 5 months in household	110/206 (53.4)	205/311 (65.9)	0.81 (0.55-1.19)	0.69 (0.50-0.96)
**Nights away from home in last month**			***<0.0001***	***<0.0001***
None	2385/3557 (67.1)	3604/4478 (80.5)	1.00	1.00
1-7	21/43 (48.8)	41/52 (78.9)	0.35 (0.17-0.72)	0.87 (0.40-1.89)
8+	65/170 (38.2)	86/173 (49.7)	0.30 (0.19-0.46)	0.37 (0.24-0.55)

Among men survey participation by those aged 18–24 years was 55.2% of those eligible and this proportion increased in each subsequent age group to reach 77.4% in those aged ≥ 55 years. The odds of survey participation among men in this oldest age group were twice those of men in the youngest (aOR 2.02, 95%CI 1.43-2.84). Among women, survey participation among those aged 18–24 years was 67.6% of those eligible, rising to 87.9% of those aged 35–44 years before declining to 81.0% of those aged ≥ 55 years. The odds of survey participation by all women aged ≥ 25 years were higher than those in women aged 18–24.

No association was shown between survey participation and religion for either men or women, and for men there was also no evidence of an association between survey participation and higher levels of education. Women who reached secondary education had the same odds of participation as women with primary education or less, but for women with a post-secondary education these odds of participation fell by 48% (aOR 0.52, 95%CI 0.38-0.70, p < 0.0001). For men and women there was evidence that being currently or formerly married were both associated with increased odds of survey participation, when compared to those who had never married. The differences were most pronounced among divorced or widowed men (aOR 2.77, 95% CI 1.93-3.97) and currently married women (aOR 2.20, 95% CI 1.71-2.84).

Among both men and women, the largest proportions of those taking part in the survey had been normally resident in their households during the year leading up to the census. The multivariate analysis showed that for men there was not a significant association between time spent away from the household and subsequent participation in the survey. In contrast, women who spent periods of time away from the household were around 30% less likely to take part in the subsequent survey, although this did not appear to be substantially influenced by the length of time away. Nights away from home in the month before the census reduced subsequent survey participation for both men and women. Men who spent up to seven nights away had odds of survey participation that were 65% lower than those of men who spent no time away (aOR 0.35, 95% CI 0.17-0.72) and this effect strengthened with increasing absence. For women, time away from home for up to seven nights in the month before the census was not associated with reduced survey participation, but those who were away for eight or more nights had odds of participation that were 63% lower than those who were never away (aOR 0.37, 95% CI 0.25-0.35).

### Self-reported knowledge of HIV status

1,491 of the men (60.3%) and 2,660 (71.3%) of the women who volunteered to take part in the medical survey already knew their HIV status. In a multivariate analysis, with all variables adjusted for each other, the odds of women knowing their HIV status were 47% higher than those of men (aOR 1.47, 95% CI 1.29-1.68, p < 0.0001). Table 2 shows, for men and women separately, the associations between the demographic and behavioural exposures measured in the survey and the adjusted association of each one with knowledge of HIV status.

**Table 2:** Self-reported knowledge of HIV status.

	Knew HIV Status/Total Participated N (%)		*Adjusted Odds Ratio p-value (LRT)* Adjusted Odds Ratio	
Exposures stratified by sex	Men	Women	Men	Women
**Age group**			***<0.0001***	***<0.0001***
18 – 24	278/525 (53.0)	570/752 (75.8)	1.00	1.00
25 – 34	325/506 (64.2)	648/778 (83.3)	1.09 (0.76-1.56)	1.18 (0.87-1.59)
35 – 44	303/441 (68.7)	602/777 (77.5)	1.29 (0.86-1.95)	0.83 (0.61-1.13)
45 – 54	305/455 (67.0)	397/586 (67.8)	1.33 (0.88-2.03)	0.51 (0.37-0.71)
55+	280/544 (51.5)	443/838 (52.9)	0.60 (0.40-0.92)	0.26 (0.19-0.37)
**Religion**			***0.1316***	***0.1891***
Christian	1114/1862 (59.8)	1982/2779 (71.3)	1.00	1.00
Muslim	377/609 (61.9)	678/952 (71.2)	0.82 (0.63-1.06)	0.88 (0.72-1.07)
**Highest level of education**			***0.0193***	***0.0108***
None/primary	940/1620 (58.0)	1681/2453 (68.5)	1.00	1.00
Secondary	417/662 (63.0)	795/1050 (75.7)	1.24 (0.99-1.56)	1.14 (0.94-1.39)
Post-secondary	134/189 (70.9)	184/228 (80.7)	1.59 (1.08-2.35)	1.74 (1.19-2.56)
**Marital status**			***<0.0001***	***<0.0001***
Never married	334/664 (50.3)	369/549 (67.2)	1.00	1.00
Currently married	948/1418 (66.9)	1549/2034 (76.2)	2.80 (1.97-3.98)	2.33 (1.75-3.08)
Divorced or widowed	209/389 (53.7)	742/1148 (64.6)	1.96 (1.30-2.93)	2.33 (1.68-3.21)
**Time spent in household last year**			***0.4577***	***0.0077***
Normally resident	1213/2028 (59.8)	2120/3046 (69.6)	1.00	1.00
6 to 12 months in household	213/333 (64.0)	394/480 (82.1)	1.19 (0.89-1.59)	1.51 (1.14-1.98)
0 to 5 months in household	65/110 (59.1)	146/205 (71.2)	0.94 (0.57-1.54)	0.91 (0.63-1.33)
**Nights away from home in last month**			***0.4289***	***0.2634***
None	1430/2385 (60.0)	2576/3604 (71.5)	1.00	1.00
1-7	16/21 (76.2)	26/41 (63.4)	1.86 (0.58-5.94)	0.62 (0.31-1.29)
8+	45/65 (69.2)	58/86 (67.4)	1.28 (0.65-2.50)	0.74 (0.42-1.28)
**Current smoker**			***0.0020***	**0.0188**
No	1273/2043 (62.3)	2589/3607 (71.8)	1.00	1.00
Yes	218/428 (50.9)	71/124 (57.3)	0.65 (0.50-0.86)	0.61 (0.40-0.92)
**Alcohol consumption**			***0.0001***	**0.2090**
Never drunk alcohol	750/1163 (64.5)	1741/2368 (73.5)	1.00	1.00
Former drinker (>12 months ago)	99/188 (52.7)	292/446 (65.5)	0.54 (0.36-0.80)	0.88 (0.68-1.14)
Current drinker (within last 12 months)	642/1120 (57.3)	627/917 (68.4)	0.61 (0.46-0.78)	0.84 (0.68-1.03)

Among those aged 18–24 years, 53.0% of men and 75.8% of women knew their HIV status. For men this proportion reached a maximum of 68.7% in those aged 35–44 years before falling back to 51.5% among those aged ≥55 years. For women the proportion rose to a maximum of 83.3% among those aged 25–34 years before falling to 52.9% among those aged ≥55 years. In the two oldest age groups the proportions of men and women knowing their HIV status were broadly the same. The adjusted odds of men aged ≥55 years knowing their HIV status was 40% lower than those among men aged 18–24 (aOR 0.60, 95% CI 0.40-0.92), whereas for women ≥55 years these adjusted odds were 74% lower when compared to women aged 18–24 (aOR 0.26, 95% CI 0.19-0.37).

The analysis found no evidence for either sex that knowledge of HIV status was associated with religion but it did show an association with education. The adjusted odds of those with post-secondary education knowing their HIV status were 59% higher among men and 74% higher among women when compared to those with primary education or less (aOR 1.59, 95% CI 1.08-2.35, p = 0.0193 and aOR 1.74, 95% CI 1.19-2.56, p = 0.0108 respectively). Being currently or formerly married were also associated with increased knowledge of HIV status for both men and women, when compared to those who had never married.

The association between knowledge of HIV status and time spent away from the household in the last year appeared to differ between men and women. For men there is no evidence of an association regardless of time spent away from home, whereas for women the adjusted odds of knowing HIV status is 51% greater among those who are away from home for up to six months when compared to those who are normally resident (aOR 1.51, 95%CI 1.14-1.98). However, for both sexes there is no evidence that nights spent away from home in the month prior to the survey had any association with knowledge of HIV status.

Smoking tobacco products was associated with lower knowledge of HIV status for both men and women. The odds of male smokers knowing their HIV status were 35% lower than those in male non-smokers (aOR 0.65, 95% CI 0.50-0.86, p = 0.0020) whereas for women the equivalent odds were 39% lower (aOR 0.61, 95% CI 0.40-0.92, p = 0.0188). Alcohol consumption was also associated with reduced knowledge of HIV status among men, with the odds of former drinkers and current drinkers knowing their status being 46% and 39% lower respectively (aOR 0.54, 95% CI 0.36-0.80 and aOR 0.61, 95% CI 0.46-0.78, p = 0.0001). However, this analysis did not show a significant association among women between alcohol consumption and knowledge of HIV status.

### Self-reported HIV status and results of survey HIV testing

All 6,202 survey participants in this analysis consented to having an HIV rapid test and of these 6,198 (2,469 men and 3,729 women) had a test result recorded. Table 3 shows that, of the 980 men who did not know their HIV status when interviewed, 979 now had an HIV test result and 24 of these (2.5%) were positive. Of the 1071 women who did not know their HIV status, 1070 now had an HIV test result of which 29 (2.7%) were positive. Of 1,321 men who reported being HIV negative at interview, 9 (0.7%) now had a positive result, whereas for the 2,311 women who self-reported as HIV negative, 18 (0.8%) now had a positive result. Four participants – one man and three women – had self-reported as living with HIV but now received a negative test result.

**Table 3:** Self-reported HIV status compared to HIV test results.

	Men				Women			
	Self-reported HIV status				Self-reported HIV status			
Survey test result	HIV positive	HIV negative	Status unknown	Men Total	HIV positive	HIV negative	Status unknown	Women Total
**HIV** **positive**	168	9	24	**201**	346	18	29	**393**
**HIV** **negative**	1	1312	955	**2268**	3	2292	1041	**3336**
**Invalid result**	1	0	1	**2**	0	1	1	**2**
**Total**	**170**	**1321**	**980**	**2471**	**349**	**2311**	**1071**	**3731**

Of the 201 men who received a positive HIV test result during the survey, 168 (83.6%) already knew that they were living with HIV. Of the 393 women who received a positive HIV result, 346 (88.0%) already knew their HIV status. Overall HIV prevalence among survey participants was 8.1% among men and 10.5% among women. 167 of the 168 men who knew that they were living with HIV reported that they were taking ART, whereas for women 342 of the 346 who knew they were living with HIV were taking ART.

## Discussion

This analysis showed that, in a cross-sectional individual-level survey conducted between 2019 and 2021 of over 8,000 adults in a rural HDSS population in Uganda, almost 40% of men and 29% of women had never previously had an HIV test. Of these, all consented to taking an HIV test as part of their survey participation and 2.5% of the men and 2.7% of the women who had never previously tested received a positive result. Among all of those living with HIV, 83% of men and 88% of women already knew their status prior to the survey, and of these 99% of both men and women self-reported that they were taking ART.

Results released by the Uganda AIDS Commission, based on national data collected to the end of 2024, report that 93% of adult men and 96% of women knew that they were living with HIV. Of these, 87% of men and 91% of women were taking ART [24]. These data included those aged 15–49 years and present national estimates in a country with substantial variations in HIV prevalence and service provision. Compared to the national averages those living with HIV in this survey had lower knowledge of their HIV status but greater uptake of ART, and this may reflect both the demographic composition of the participants and their ease of access to several Ministry of Health clinics that have made ART available without charge.

Knowledge of HIV status varied by both sex and age. 75% of the youngest women in the survey knew their status compared to 53% of men, but by the time both sexes were aged 45 years and above this difference had disappeared, with just over half of men and women knowing their status. These associations between age, sex and knowledge of HIV status may reflect the epidemiology of HIV in a setting that has experienced a generalised epidemic for over four decades. The first cases of AIDS were identified in Uganda in 1982 [23] but voluntary counselling and testing for HIV was not introduced in the country until 1990 and the distribution of effective ART did not begin until 2004 [25]. Whilst dates of birth may not be precise, survey participants aged 45 and above at the time of interview were likely to have experienced young adulthood during a time when HIV in Uganda was either unidentified or untreatable. Older survey participants may have seen contemporaries of similar age die of AIDS-related illnesses and may have plausibly attributed their own survival to an absence of HIV infection. If their self-perception of HIV risk remained low this may have created a continued avoidance of testing among older GPC residents, even after it had become widely available. This may help to explain the similarity in the proportions of older men and women who did not know their HIV status, and also why the HIV prevalences among those who had never previously tested were low when compared to prevalences in the full survey population. Nevertheless these were sufficient to impact the first UNAIDS target, the percentages of those living with HIV who were aware of their diagnosis.

Other studies in East African settings have identified similar demographic characteristics regarding hesitancy to test for HIV. An analysis of Demographic and Health Survey data from 16 sub-Saharan African countries showed that, in Uganda in 2011, 69% of all those surveyed had previously tested for HIV [14], comparable to the 66.9% identified in this analysis. The reluctance of men to test for HIV has been well documented [26] and in Uganda the effects of stigma and concepts of masculinity [21] continue to act as a deterrent to men of all ages. Lower knowledge of HIV status has also been reported among older people across eight sub-Saharan African countries [27]. The introduction of routine HIV testing into antenatal care under the Option B+ initiative that was introduced in Uganda in 2012 [28] may help to explain the higher proportions of the youngest women who knew their HIV status when compared to the youngest men. The findings of this study that reduced knowledge of HIV status was independently associated with lower formal education, never being married and consuming alcohol have also been identified in other studies [14,29,30].

Overall HIV prevalence in these survey participants was 9.6%. This is very close to the prevalence that the Uganda AIDS Commission reported for the Kalungu district within which the GPC lies [24]. Southwest Uganda has among the highest prevalence of HIV in the country [24] which is reflective of both the high incidence of HIV in the past and the high uptake of ART that has increased life expectancy and correspondingly raised prevalence. As with other parts of Eastern and Southern Africa, HIV incidence has been falling steadily throughout Uganda in the last decade as the protective effect of ART reduces transmission [31,32]. The HIV prevalence among women respondents in the survey was 10.5%, compared to 8.1% among men. The increased risk of HIV acquisition among women is widely recognised and was first reported in this cohort in 1994 [33].

Linking the census and survey records enabled potential selection bias to be revealed that could arise from voluntary participation in the medical survey. Survey participation was consistently lower among men when compared to women, with the lowest participation among the youngest men. Participation was also lowest among those who had never married. As men and those who had never married were also among those less likely to know their HIV status, the results for knowledge of status for these groups are likely to be an underestimate and help to highlight those who should be prioritised in order to minimise the potential risk posed by undiagnosed HIV infection.

A key strength of this analysis is that it was based on data from a large population study in which HIV testing was part of the study protocol, so that virological status was based on direct measurements. An established research environment and consistent fieldwork over a period of two years also helped to maximise resident participation and data consistency. Limitations are the absence of data on sexual behaviour, and data on HIV viral load that would have enabled reporting of the viral suppression component of the UNAIDS targets. Nevertheless this study demonstrates the value of direct estimation of HIV testing through population surveys as a means of understanding where the threat posed by untreated HIV infection may continue to lie.

The HIV prevalences in this Cohort means that it is heavily reliant upon sustained distribution of sufficient ART. Since 2004 the cornerstone of access to ART in Uganda has been the United States President's Emergency Plan for AIDS Relief (PEPFAR). Uganda was the first country in the world to provide PEPFAR-supported ART and the Plan has been credited with averting 600,000 HIV-related deaths and 500,000 new infections across the country [34]. In 2023 it was estimated that 93% of the 1.4 million Ugandans living with HIV were receiving PEPFAR-supported ART [34]. In early 2025 the global functioning of PEPFAR was disrupted by the change in political administration in the USA. On 24th January 2025 all funding for PEPFAR was frozen, including that of existing grants and contracts [35]. On 1^st^ February a limited waiver was granted to enable PEPFAR to continue “life-saving HIV care and treatment services” that included the provision of ART [36], but by mid-March 2025 UNAIDS reported that, while stocks of ART were available in Uganda, distribution had been disrupted, all facilities were operating at reduced capacity and some non-Governmental and community-run organisations had ceased to provide services [37].

In September 2025 the US Department of State published the America First Global Health Strategy [38]. While this retains many of the objectives of the 2021 Political Declaration and has received a supportive response from UNAIDS [39] its emphasis upon supported countries transitioning fully to “self-reliance” does not give country-specific details. It has been reported that Uganda may be required to be fully self-sufficient in funding ART within the next five to eight years [40], potentially a substantial challenge for a country with a sustained and generalised HIV epidemic that has been primarily addressed through external funding across two decades.

In February 2025 PEPFAR was also required to cease funding of all HIV prevention for the key populations that the 2021 declaration on AIDS had explicitly prioritised [41]. This is despite 80% of new global HIV acquisition, and 25% of new acquisitions in sub-Saharan Africa, occurring among key populations in 2022 [42]. Furthermore the stigmatisation of these populations within Uganda can be widespread and severe [43,44], including sentences of life-imprisonment and, in some cases, the death penalty for consensual sex between men [45]. This legislation, passed in 2023 by 387 of 389 Ugandan MPs, caused The Global Fund, UNAIDS and PEPFAR to respond with a joint statement concluding that “Uganda’s progress on HIV response is now in grave jeopardy” [46].

People living with HIV in Uganda therefore face a variety of pressures that can hinder learning their HIV status and taking steps to manage it. Population surveys such as that described here should continue to have a key role in describing progress and barriers toward reaching global HIV management targets during periods that can be characterised by rapid, unexpected and comprehensive change.

## Supporting information

S1 Checklist Inclusivity in Global Research questionnaire. (DOCX)
