# Supplementary material for: Knowledge of HIV status in a rural Ugandan population with a sustained, generalised HIV epidemic
Source: PLOS Glob Public Health. 2026 Oct 1;6(10):e0007354. doi: 10.1371/journal.pgph.0007354 (PMC13630239; doi:10.1371/journal.pgph.0007354)
Supplement: Supplementary file 2 [file pgph.0007354.s002.docx]

**Response to original review**

**Reviewer comment: The overall aim of this study is either missing or not clear. Authors need to present why this survey was done and make sure they align to that all the way to the results and discussion.**

*Author response: The primary aim and content of the article has been clarified to measurement of the knowledge of HIV status among this rural Ugandan population, through asking for self-report of HIV status before offering an HIV test. Through this approach the study was able to determine a) the self-reported knowledge of HIV status in the population; b) the proportion of the population living with HIV that already knew its HIV status (the “first 95” of the UNAIDS targets); and c) demographic and behavioural exposures that may be associated with knowledge of HIV status. Secondary aims of the study were to update the HIV prevalence in the population and to determine, among those who had self-reported living with HIV, the proportion taking ART. These aims are given in lines* ***109 to 117*** *of the tracked-changes document.*

**Reviewer comment: The title of this paper has aspects like HIV testing, prevalence, risk factors and attainment of UNAIDS HIV treatment targets. Yet, in the results section we see results on the odds of survey participation (unrelated in my honest opinion), risk factors of living with HIV (confusing as opposed to odds of knowledge of HIV status) yet say absolutely nothing about treatment adherence/retention and viral suppression.**

*Author response: The title of the paper has been changed to “Knowledge of HIV status in a rural Ugandan population with a sustained generalised HIV epidemic”. The design of the study was that a census was conducted of all residents of a rural HDSS and these census data then formed the study population for a medical survey in which participants volunteered to take part. The analysis of survey participation was to reveal any selection biases that may arise from such voluntary survey participation, and for this reason it has been retained. However the explanation of the study design has been expanded upon in the Methods section in lines* ***157 to 177*** *and* ***196 to 199*** *of the tracked-changes document. As the analysis of risk factors for HIV acquisition was felt to be confusing, this has been removed from the manuscript.*

**Reviewer comment: Moreso, given the study aim of assessing attainment of UNAIDS treatment targets, I was expecting to see performance of the GPC with respect to the 95-95-95 overall or for men and women separately (substituting viral load with CD4 count doesn’t provide any element of response to the second and third 95).**

*Author response: Use of CD4 count as a proxy measure for viral suppression has been removed from the manuscript. The article now focuses primarily on assessing knowledge of HIV status, and on the proportion of those living with HIV who already knew their status. The analysis does include the proportion of this group that self-reported taking ART but it does not describe this in terms of the UNAIDS targets.*

**Reviewer comment: Authors talk about “exposure” and “risk factors” (instead of “factors associated”) meanwhile presenting odd ratios. Given authors talked of a cohort, I was expecting multiple follow up over time thus risk and/or survival analysis.**

*Author response: Whilst the study population is named the General Population Cohort because of its longitudinal structure, this analysis only used a single round of census data to define the study population. The subsequent medical survey was also a single round and so as both datasets were cross-sectional, odds ratios were used to measure effect. These analytical approaches have been explained in more detail in the Methods section, in lines* ***202 to 206*** *of the tracked-changes document.*

**Reviewer comment: Under study setting and population, I was expecting to get more information on the 25 villages (why these villages?) and the study populations included in the study, not the surveillance system (GPC).**

*Author response: Some more explanation has been given as to why these villages have been the focus of the epidemiological study of HIV, in lines* ***144 to 147*** *of the tracked-changes document. In this case the study population and the surveillance system are the same thing i.e. an attempt was made to interview all residents of the HDSS in order to obtain the study population. This has been clarified in the Methods section.*

**Reviewer comment: Also, it’s mentioned that medical survey was conducted from the GPC meaning that the routine surveillance data were not used. The question now becomes how were the households sampled? I acknowledge authors said all but how did they make sure of that given we don’t have the baseline numbers they started with.**

*Author response: The study population was derived from a new census of the 25 villages that comprise the General Population Cohort. These villages have been selected to be representative of a rural East African community. All GPC households were included in the census and so there was no household sampling. The census provided the study denominators and eligible household residents were then invited from the census population to participate in the medical survey on a voluntary basis. Survey participation was analysed to reveal any selection biases arising from voluntary participation. These points have been clarified in the Methods section of the manuscript.*

**Reviewer comment: At certain points in the paper, authors leave the impression that this was an analysis pulled from already available data while at other points it seems like it was a conducted study with aim predetermined before data collection. This should be clarified.**

*Author response: All data used in the study (census and medical survey) were newly collected between 2019 and 2021, and the aims of the analysis were determined before it was carried out. This point has been clarified in the Methods section.*
