# Supplementary material for: Knowledge of HIV status in a rural Ugandan population with a sustained, generalised HIV epidemic
Source: PLOS Glob Public Health. 2026 Oct 1;6(10):e0007354. doi: 10.1371/journal.pgph.0007354 (PMC13630239; doi:10.1371/journal.pgph.0007354)
Supplement: Supplementary file 3 [file pgph.0007354.s003.docx]

**Response to second review**

**Reviewer comment: My only remaining concerns are with the abstract:**

- **While the authors revised the main text to clarify the objectives of the study, the Abstract does not include explicit and clear objectives. Please update the Abstract with the specific objectives of the study.**
- **The concluding sentences of the Abstract should be updated to reflect the interpretation of the main objectives. For example, did the study find that the UNAIDS target was met?**

*Author response: The abstract has been revised as suggested. The opening paragraph now describes the primary and secondary objectives of the study, following the same sequence and terminology as those given in the closing paragraph of the Introduction. These changes are shown in lines* ***29 to 35*** *of the tracked-changes document. The last paragraph of the abstract has also been edited to more fully reflect the interpretation of these objectives. These points are made in the same order as the objectives at the start of the abstract and also refer to the UNAIDS testing targets. These changes are shown in lines* ***52 to 59*** *of the tracked-changes document. No other parts of the manuscript have been changed.*
